# Predictors of Intravenous Immunoglobulin Non‐Responsiveness in Children With Kawasaki Disease

**DOI:** 10.1002/iid3.70354

**Published:** 2026-02-12

**Authors:** Yun Cheng, Biquan Chen

**Affiliations:** ^1^ Department of Infection Diseases Anhui Provincial Children's Hospital Hefei Anhui China

**Keywords:** children, intravenous immunoglobulin non‐responsiveness, Kawasaki disease, systemic immune‐inflammation index

## Abstract

**Background:**

This retrospective study assesses whether the systemic immune‐inflammation index (SII) is a better predictor of intravenous immunoglobulin (IVIG) non‐responsiveness (IVIG‐NR) in children with Kawasaki disease (KD children) than the neutrophil‐to‐lymphocyte ratio (NLR) and platelet‐to‐lymphocyte ratio (PLR).

**Methods:**

After 458 pediatric KD patients were categorized as IVIG responders or non‐responders, blood test data were collected from both groups prior to IVIG administration. Upon SII, NLR, and PLR calculation, IVIG‐NR predictive performances of these markers were evaluated through receiver operating characteristic curve evaluation, and IVIG‐NR risk factors (RFs) were determined by multivariate logistic regression.

**Results:**

Compared to IVIG responders, the non‐responders showed significantly higher NLR, PLR, and SII (*p*‐values < 0.05). The cut‐off values of these markers for optimal IVIG‐NR prediction were 4.245, 147.736, and 1465.238, respectively.

**Conclusion:**

Although all three of them were the independent RFs for IVIG‐NR in KD children, SII was a better IVIG‐NR predictor than NLR and PLR.

## Graphical abstract

Compared to IVIG responders, the non‐responders showed significantly higher NLR, PLR, and SII (*p*‐values < 0.05). SII was a better IVIG‐NR predictor than NLR and PLR.

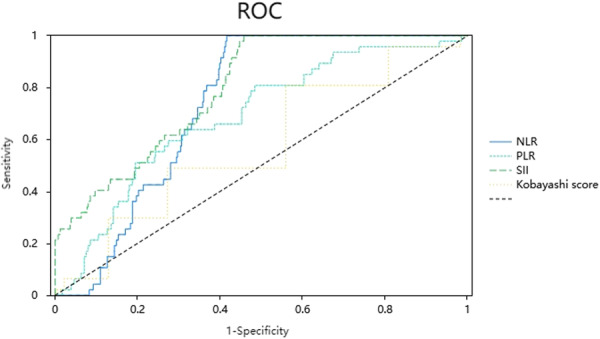

## 1 Introduction

Given that coronary artery damage is its most serious complication, Kawasaki disease (KD)—a systemic disorder characterized by acute fever, systemic vasculitis, and rash—is one of the leading causes of acquired heart disease in pediatric patients [1]. Predominantly affecting children under five, it can generally be treated effectively through prompt medication with intravenous immunoglobulin (IVIG) and aspirin [2]. However, IVIG non‐responsiveness (IVIG‐NR) [1, 3], which elevates coronary artery damage risks [1], occurs in 10–20% of KD patients.

Currently, IVIG‐NR remains a clinical diagnosis, as no diagnostic markers possess sufficient sensitivity and specificity. IVIG‐NR in KD patients has been predicted via risk‐scoring systems utilizing commonly used laboratory data. The systems developed by Kobayashi et al. [4], Egami et al. [5], and Sano et al. [6] are widely used in Japan, but they are less accurate for non‐Japanese populations [7]; the predictive accuracy of existing risk‐scoring systems is influenced by ethnicity and geographical variations. As a result, these systems have not yielded satisfactory outcomes when applied to diverse populations.

In KD patients who are unresponsive to immunoglobulin, initial treatment with a corticosteroid‐IVIG combination is more effective than that with IVIG alone in preventing coronary artery damage [8]. Given this challenge, identifying a universal marker for IVIG‐NR in KD children is an urgent task. The early detection of IVIG resistance is crucial for optimizing treatment strategies and improving prognosis in pediatric KD patients.

As classical inflammatory markers, neutrophils are crucial to the immune response during the first KD pathological stage. In the second KD pathological stage, anti‐inflammatory cytokines are released, and they induce immunosuppression, leading to lymphocyte apoptosis [9]. Therefore, higher neutrophil and lower lymphocyte counts may indicate that the patient is experiencing a severe inflammatory response and the active clinical course of KD. In addition, platelet counts may also be elevated due to proinflammatory cytokine‐stimulated megakaryocyte proliferation, reflecting inflammation. Previous studies have demonstrated associations between IVIG resistance in KD and various blood parameters, including neutrophils, lymphocytes, platelets, the neutrophil‐to‐lymphocyte ratio (NLR), and the platelet‐to‐lymphocyte ratio (PLR). However, since KD is a complex systemic vasculitis, these markers, which are derived from one or two routine blood tests, may not fully capture the inflammatory processes underlying KD.

Recent studies have identified novel inflammatory markers related to immune‐mediated diseases [10, 11, 12], including the systemic immune‐inflammation index (SII = platelet count × neutrophil count/lymphocyte count), the systemic inflammation response index (monocyte count × neutrophil count/lymphocyte count), and the pan‐immune‐inflammation value (monocyte count × platelet count × neutrophil count/lymphocyte count). However, there are few studies on the IVIG‐NR predictive performance of SII.

Therefore, this retrospective study explores the association of SII with IVIG‐NR in children with KD (KD children).

## 2 Materials and Methods

### 2.1 Data Collection

This study involved 458 newly diagnosed KD patients treated at Anhui Provincial Children's Hospital (APCH) in the period between January 2022 and December 2023, consisting of 411 IVIG responders and 47 IVIG non‐responders. Their KD diagnosis was based on the 2017 American Heart Association guidelines [1]: KD was indicated by a fever lasting for 5 days or more along with four or more of the five main KD clinical features: cervical lymphadenopathy, extremity alterations, rash, oral alterations, and bilateral conjunctival injection without exudates.

IVIG‐NR was characterized by a body temperature above 38°C persisting for more than 36 h after IVIG treatment or a fever recurring within 2 weeks (typically 2 to 7 days) post‐treatment, along with at least one major KD clinical feature. Within 10 days of KD onset, patients received standard treatment consisting of IVIG (2 g/kg) and oral aspirin (30–50 mg/kg). When coronary artery dilation was absent, patients were treated with a low dose (3–5 mg/kg) of aspirin for 6–8 weeks.

We excluded patients with systemic autoimmune diseases, infectious diseases, metabolic or hematologic disorders, tumors, renal diseases, other cardiac conditions, or malnutrition; prior use of glucocorticoids, immunosuppressive drugs, or IVIG before admission; or incomplete laboratory data during hospitalization.

Before IVIG treatment, blood test data, including the total white blood cell count (WBCC), neutrophil count (NTPC), lymphocyte count (LMPC), and platelet count (PLTC), were collected for NLR, PLR, and SII calculation. Additionally, general information such as gender, age, and illness duration before IVIG initiation was recorded. Patients were grouped according to their response to IVIG, and univariate analysis was conducted. Statistically significant variables were then analyzed with multivariate logistic regression, and the risk factors (RFs) for IVIG‐NR in KD children were identified.

### 2.2 Statistical Analysis

SPSS 23 (IBM) was employed for statistical analysis. On the one hand, continuous and categorical variables were presented as means ± standard deviations and percentages, respectively. On the other hand, they were evaluated using the independent samples *t*‐test and chi‐square test, respectively. The RFs for IVIG‐NR in KD children were identified by multivariate logistic regression. The NLR, PLR, and SII cut‐off values for optimal IVIG‐NR prediction were determined using ROC curves. Statistical significance was represented by *p*‐values < 0.05.

## 3 Results

### 3.1 Clinical Characteristics of IVIG Responders and Non‐Responders

This study included 458 patients categorized as IVIG responders or non‐responders, consisting of 166 (36.23%) males and 293 (63.97%) females (Table 1). Among these patients, 47 (10.26%) were IVIG non‐responders, including 18 males and 29 females. No significant differences in age distribution (22.31 ± 9.59 months vs. 23.78 ± 10.32 months, *p* = 0.322), gender distribution (36.01% vs. 38.30% for males and 63.99% vs. 61.70% for females, *p* = 0.757), or illness duration before IVIG initiation (5.02 ± 0.47 days vs. 4.97 ± 0.44 days, *p* = 0.51) were observed between the groups.

**TABLE 1:** Characteristics of IVIG responders and non‐responders.

Parameter		IVIG Non‐responders (*n* = 47)	IVIG Responders (*n* = 411)	*t*/*χ* ^2^	*p*
Age/month		23.78 ± 10.32	22.31 ± 9.59	0.992	0.322
Duration of illness before IVIG initiation		4.97 ± 0.44	5.02 ± 0.47	−0.659	0.51
NTPC		10.69 ± 1.84	9.99 ± 2.30	2.000	0.046*
LMPC		2.42 ± 0.39	2.39 ± 0.58	0.476	0.636
WBCC		14.78 ± 6.20	15.12 ± 4.80	−0.36	0.72
CRP		75.22 ± 29.70	62.40 ± 29.32	2.835	0.005**
NLR		4.42 ± 0.26	4.21 ± 0.39	4.851	0.000**
PLTC		379.98 ± 41.73	343.80 ± 70.45	5.162	0.000**
PLR		159.14 ± 17.79	146.52 ± 20.03	4.134	0.000**
SII		1678.43 ± 207.56	1438.88 ± 277.94	7.208	0.000**
Sex	Male	18 (38.30)	148 (36.01)	0.096	0.757
	Female	29 (61.70)	263 (63.99)		
Kobayashi score		5.000 (5.0, 7.0)	5.000 (4.0, 6.0)	−3.929	0.000**
Egami score		2.000 (2.0, 3.0)	2.000 (1.0, 2.0)	−3.383	0.001**

* *p*< 0.05

** *p*< 0.01.

*Note:* Presented data are means ± standard deviations.

Abbreviations: CRP, C‐reactive protein; IVIG, intravenous immunoglobulin; KD, Kawasaki disease; LMPC, lymphocyte count; NTPC, neutrophil count; NLR, neutrophil‐to‐lymphocyte ratio; PLR, platelet‐to‐lymphocyte ratio; PLTC, platelet count; SII, systemic immune‐inflammation index; and WBCC, white blood cell count.

Compared to IVIG responders, the non‐responders exhibited significantly higher levels of SII, NLR, PLR, NTPC, C‐reactive protein (CRP), and PLTC. However, the groups did not differ significantly in LMPC or WBCC (*p*‐values > 0.05).

### 3.2 Predictive Performance of Clinical Indicators for IVIG‐NR in KD Children

**FIGURE 1:**
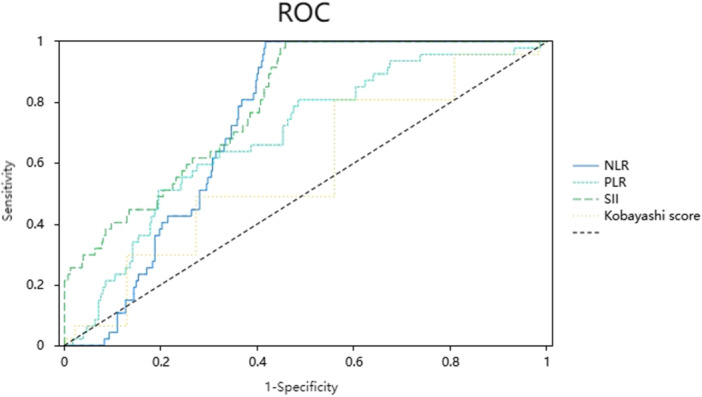
ROC curves of clinical predictors for IVIG‐NR in KD children. KD, Kawasaki disease; IVIG‐NR, intravenous immunoglobulin‐non‐responsiveness; ROC, receiver operating characteristic.

As shown in Figure 1 and Table 2, the areas under the receiver operating characteristic (ROC) curves (AUCs) for Kobayashi score, CRP, NLR, PLR, and SII are 0.671, 0.629, 0.706, 0.685, and 0.747, respectively. The same table indicates that the cut‐off values of these markers for optimal IVIG‐NR prediction are 479.44, 4.245, 147.736, and 1465.238, respectively.

**TABLE 2:** Predictive performances of clinical indicators for IVIG‐NR in KD children.

Item	Cut‐off	Sensitivity	Specificity	AUC	95% CI
CRP	79.44	0.574	0.72	0.629	0.542–0.716
NLR	4.245	0.957	0.584	0.706	0.647–0.766
PLR	147.736	0.809	0.516	0.685	0.607–0.762
SII	1465.238	0.936	0.543	0.747	0.684–0.811
Kobayashi score	4	0.809	0.44	0.671	0.594–0.748

Abbreviations: AUC, area under a curve; CI, confidence interval; CRP, C‐reactive protein; IVIG, intravenous immunoglobulin; KD, Kawasaki disease; NLR, neutrophil‐to‐lymphocyte ratio; PLR, platelet‐to‐lymphocyte ratio; SII, systemic immune‐inflammation index.

### 3.3 Multivariate Analysis of Factors Related to IVIG‐NR in KD Children

According to multiple logistic regression, the following were the independent RFs for IVIG‐NR in KD children: CRP ≥ 79.44 mg/L, NLR ≥ 4.245, PLR ≥ 147.736, and SII ≥ 1465.238 (Table 3; *p*‐values < 0.05). To avoid collinearity, NTPC and PLTC, from which NLR, PLR, and SII are derived and correlated, were excluded from the regression analysis.

**TABLE 3:** Predictors of IVIG‐NR in KD children.

Item	Regression coefficient	Standard error	*Z*‐Value	Wald *χ* ^2^	*p*‐Value	OR	OR 95% CI
CRP	0.013	0.006	2.274	5.169	0.023	1.013	1.002–1.024
NLR	1.132	0.462	2.450	6.002	0.014	3.102	1.254–7.672
PLR	0.027	0.009	2.988	8.930	0.003	1.028	1.009–1.046
SII	0.003	0.001	4.626	21.397	0.000	1.003	1.002–1.005
Kobayashi score	0.346	0.168	2.054	4.221	0.04	1.414	1.016–1.967
Egami score	0.289	0.337	0.857	0.735	0.391	1.335	0.690–2.582

Abbreviations: CI, confidence interval; CRP, C‐reactive protein; IVIG, intravenous immunoglobulin; KD, Kawasaki disease; NLR, neutrophil‐to‐lymphocyte ratio; OR, odds ratio; PLR, platelet‐to‐lymphocyte ratio; SII, systemic immune‐inflammation index

## 4 Discussion

According to previous studies, 10%–20% of KD patients exhibit IVIG‐NR after initial treatment, which aligns with our findings.

KD is an acute non‐specific inflammatory disorder affecting medium‐sized arteries, although its etiology remains unclear [13]. Systemic inflammatory responses are crucial to KD pathogenesis. Sano et al. [6] demonstrated that elevated NTPCs increase IVIG‐NR risks, suggesting the crucial roles of neutrophils in KD. This study similarly reported a higher NTPC in IVIG non‐responders. Neutrophil activation can cause tissue damage and endothelial cell injury through the release of elastase, myeloperoxidase, and reactive oxygen species, exacerbating systemic inflammation [14, 15, 16].

In contrast, lymphocytes play different immune regulatory roles. Excessive neutrophil activation can trigger an inflammatory cascade, disrupting the balance between inflammation and immunity, leading to environmental disturbances and extensive lymphocyte apoptosis. As lymphocytes are crucial for vascular endothelial health [17], lymphopenia is a negative prognostic indicator of acute coronary syndrome and heart failure [18, 19].

Another critical indicator of inflammatory progression is PLTC. Elevated levels of pro‐inflammatory cytokines in systemic inflammatory responses lead to megakaryocyte proliferation, which results in increased PLTCs. This induces the release of inflammatory mediators such as histamine, enhancing vascular permeability, inflammatory cell infiltration, and subsequent endothelial damage [20, 21, 22, 23]. The dynamic changes in inflammatory cells and factors lead to persistently uncontrolled inflammation, inducing systemic arterial inflammation, particularly in the coronary arteries. This causes endothelial damage, internal elastic lamina disruption, smooth muscle necrosis, vascular fibrosis, and aneurysm formation, ultimately contributing to the development of KD [1, 24].

NLR and PLR have been identified as the RFs for IVIG‐NR in KD [25, 26, 27]. Our findings corroborate the aforementioned finding, showing that IVIG non‐responders had significantly higher NLR and PLR compared to the responders. As determined by ROC curves, the cut‐off values for optimal IVIG‐NR prediction were 4.245 for NLR and 147.736 for PLR. Logistic regression analysis further confirmed that NLR and PLR were the independent RFs for IVIG‐NR in KD. These results are consistent with previous research.

SII was first introduced in 2014 to predict prognosis in cancer‐related inflammation and has since been applied to various diseases [28, 29, 30]. Compared to NLR and PLR, SII offers enhanced accuracy as an inflammation indicator because it integrates peripheral blood NTPC, LMPC, and PLTC, reflecting their interrelationships more comprehensively.

Only few studies have explored the association between SII and KD. Moreover, although Huang et al. [17] have identified SII as an important RF for CAL in KD, reports linking SII to IVIG‐NR in KD are limited. This study assessed SII alongside NLR and PLR, demonstrating that their AUCs were 0.747, 0.706, and 0.685, respectively. Sensitivity values for these markers were 93.6%, 95.7%, and 80.9%, respectively, while specificity values were 54.3%, 58.4%, and 51.6%, respectively. SII demonstrated a stronger linear correlation with IVIG resistance and a higher AUC compared to NLR and PLR. Through multiple logistic regression, the roles of SII, NLR, and PLR as the independent RFs for IVIG‐NR in KD were confirmed.

Several well‐established risk scores, such as the Kobayashi and Egami scores, have been developed to predict IVIG non‐responsiveness, primarily in Japanese populations [4, 5]. However, a recent meta‐analysis has demonstrated that these models exhibit only modest sensitivity and low positive predictive value in real‐world, multi‐ethnic settings [31], limiting their utility in guiding universal upfront intensified therapy. This variability may be attributed to differences in study design and genetic backgrounds. Although the present retrospective study identified SII, NLR, and PLR as independent predictors, we acknowledge the inherent limitations of such a design in establishing robust predictive models. To mitigate potential confounding, we employed multivariate logistic regression, adjusting for key clinical variables. Nevertheless, future prospective, multi‐center studies with larger sample sizes are warranted to validate our findings and allow for more advanced methodologies, such as propensity score analysis, to further strengthen the evidence.

Furthermore, the evolving landscape of KD diagnosis adds another layer of complexity to risk prediction. The classical requirement of 5 days of fever (AHA 2017 guidelines [1]) is being reconsidered. The recent AHA 2023 scientific statement [32] and Japanese guidelines [33] emphasize that a diagnosis can be made in the presence of coronary abnormalities even with shorter fever duration, reflecting a shift towards earlier diagnosis. This evolution may challenge the applicability of prediction models heavily reliant on clinical signs assessed at a fixed timepoint (e.g., Days 4–5 of fever). In this context, objective, quantitative hematologic indices like SII, NLR, and PLR, which can be measured at the time of presentation and are independent of fever duration or the subjective assessment of clinical signs, may offer a significant advantage. They could provide a more universally applicable tool for early risk stratification across diverse populations and evolving diagnostic criteria.

The ultimate clinical significance of predicting IVIG resistance lies in its strong association with an increased risk of coronary artery complications. While the present study focused on the initial treatment response, it is well‐established that IVIG non‐responders are at a significantly higher risk for developing coronary artery abnormalities (CAA) [1, 8]. Therefore, identifying a robust predictor like SII is a crucial first step in risk‐stratifying patients for the most serious sequelae of KD. This is further supported by a recent study by Huang et al. [17], which directly demonstrated that SII is an independent RF for CAA, strengthening the biological plausibility of our findings.

This study highlights that SII provides a significant advantage over individual markers such as NTPC, LMPC, and PLTC by offering a more comprehensive assessment of inflammation. Additionally, SII can be easily determined through routine blood tests, and there are no limitations regarding the population for its application. Although this is a single‐center retrospective study, the potential of SII as an easily accessed inflammatory marker for IVIG‐NR in KD patients is clearly demonstrated.

This study has four limitations. First, it is a single‐center retrospective study of a Chinese population, which may introduce selection bias. Secondly, our study is limited by its retrospective, single‐center design. Although we employed multivariate regression to control for identifiable confounders, residual confounding from unmeasured factors may persist. Furthermore, the sample size, particularly of the non‐responder group, limited the application of more advanced techniques like propensity score matching. We acknowledge that prospective, multi‐center studies with larger samples are necessary to validate our findings and allow for more sophisticated adjustments for potential selection bias. Third, and most importantly, this study lacks analysis of coronary artery outcomes due to the unavailability of systematic and quantifiable echocardiographic data. This precludes any direct assessment of the relationship between SII and CAA in our cohort. This is a significant limitation that underscores the need for future prospective studies designed with protocol‐driven coronary imaging at diagnosis and during long‐term follow‐up to directly validate the utility of SII for predicting the most serious complication of KD.

## 5 Conclusion

SII is a better predictor of IVIG‐NR in KD children than NLR and PLR. Thus, it can serve as a marker of IVIG‐NR in KD patients.

## Author Contributions

**Yun Cheng:** conceptualization, methodology, software, formal analysis, investigation, data curation, writing – original draft preparation, visualization, project administration. **Biquan Chen:** conceptualization, methodology, validation, writing –review and editing, supervision.

## Ethics Statement

This study was approved by the Ethics Committee of APCH and conducted in accordance with the Declaration of Helsinki. No informed consent was required as this is a retrospective study (waiver no. EYLL‐2023‐041).

## Conflicts of Interest

The authors declare no conflicts of interest.

## Data Availability

Patient data were obtained from the electronic medical record of APC.
